# Association of Maximum Troponin Levels With Diagnosis of Acute Myocardial Infarction and Elevated Risk of Mortality

**DOI:** 10.31486/toj.20.0135

**Published:** 2021

**Authors:** Jerry Fan, Kendall Hammonds, Bright Izekor, Clinton Jones, Patrick McGrade, Jeffrey B. Michel, R. Jay Widmer

**Affiliations:** ^1^ Division of Internal Medicine, Scott & White Heart Memorial Hospital, Baylor Scott & White Health, Temple, TX; ^2^ Division of Biostatistics, Scott & White Heart Memorial Hospital, Baylor Scott & White Health, Temple, TX; ^3^ Department of Internal Medicine, Division of Interventional Cardiology, Scott & White Heart Memorial Hospital, Baylor Scott & White Health, Temple, TX

**Keywords:** *Acute myocardial infarction*, *mortality*, *troponin*

## Abstract

**Background:** Cardiac troponins I and T are highly sensitive and specific markers for acute myocardial infarction (AMI). However, a wide range of non-AMI conditions can also cause significant elevations in cardiac troponins. Given the deleterious impact of misdiagnosis of AMI, the ability to risk-stratify patients who present with an elevated troponin is paramount. We hypothesized that the maximum troponin level would be more predictive of mortality and the diagnosis of AMI than the initial troponin level or change in troponin level.

**Methods:** Patient records from a 9-hospital system (n=30,173) in Texas were reviewed during a 24-month period in 2016-2017. Data collected for patients aged ≥40 years included *International Classification of Diseases, Tenth Revision* diagnoses, troponin I, demographic data (age, sex, smoking history, and chronic medical conditions), and death during hospitalization. We used logistic regression with the Firth penalized likelihood approach to determine the predictive ability of initial, maximum, and change in troponin level for mortality and the diagnosis of AMI.

**Results:** Demographic characteristics of our cohort included a median age of 70 years, with 48.05% male and 51.95% female. The most common preexisting risk factor was hypertension in 78.81% of the cohort. Notable findings from the logistic regression include the predictive ability of maximum troponin on the odds of death by 0.7% for each unit of increase in troponin value. Also, the odds of AMI increased by 3.1% for each unit of increase in the maximum troponin value.

**Conclusion:** Regardless of the level, a detectable amount of troponin in the serum results in a significantly elevated risk of mortality. Many patients with elevated troponin levels leave the hospital without a specific diagnosis, which can lead to poor outcomes because a detectable troponin does not represent a no-risk population. Our study demonstrates that maximum troponin level is a more sensitive and specific predictor of mortality than initial or change in troponin. Similarly, maximum troponin is the most predictive of AMI vs other causes of troponin elevation, likely because of the correlation between rising troponin levels and cardiomyocyte damage. Further studies are needed to correlate maximum troponin levels and clinical manifestations, which may be helpful in redefining AMI so that AMI can be distinguished more easily from non-AMI diagnoses.

## INTRODUCTION

Acute myocardial infarction (AMI) is a major cause of worldwide morbidity and mortality.^1-4^ The ability of physicians to rapidly and accurately diagnose AMI leads to improved outcomes.^1,2,5^ However, given the wide variety of symptoms associated with AMI, cardiac troponins are frequently ordered for an assortment of complaints, many of which are not AMI-related.^4,6^ The ability to distinguish between AMI and non-AMI conditions is paramount because the management is significantly different with regard to anticoagulation, antiplatelet, and hemodynamic factors.^6^

The Fourth Universal Definition of Myocardial Infarction identifies the criteria for AMI as detection of cardiac troponin above the 99th percentile of the upper reference limit with a significant rise or fall and evidence of myocardial ischemia (symptoms, electrocardiogram changes, pathological Q wave, new loss of viable myocardium, new regional wall motion abnormality, or coronary thrombus by angiography).^1,3,5,7^

Successive generations of cardiac troponins from conventional to highly sensitive have a lower limit of detection that provides an opportunity for early detection of AMI but also for non-AMI conditions.^7,8^ Studies have suggested that an increase of 20% for conventional or 30% for sensitive cardiac troponin assays should be added to the Fourth Universal Definition of Myocardial Infarction criteria because these increases separate an acute elevation from background elevations, thereby allowing for further discrimination between acute and chronic causes of troponin elevation.^7,8^ In general, an acute change in troponin level is associated with AMI, while a consistent elevation is associated with non-AMI causes.^1^ Regardless of the cause, mortality increases with elevations in troponin.^6^ For this study, we attempted to determine if the initial troponin, change in troponin, or maximum troponin level is diagnostic of AMI and mortality.

## METHODS

The population in this analysis consisted of 77,408 unique patients (based on medical record number) who presented to a 9-hospital system in Texas during 2016-2017. Patients younger than 40 years and patients without troponin tests were excluded, leaving a cohort of 30,173 patients who had at least 1 troponin test and were ≥40 years. The primary *International Classification of Diseases, Tenth Revision* diagnoses at discharge, troponin I test data, baseline characteristics and risk factors, and death during index admission for each patient were collected. Institutional review board approval was obtained for this study under an expedited review as it involved no greater than minimal risk to subjects.

Baseline characteristics and risk factors included age, sex, smoking history, hypertension, hyperlipidemia, diabetes mellitus, coronary artery disease, chronic kidney disease including end-stage renal disease, chronic obstructive pulmonary disease, prior cerebrovascular accident, and prior AMI.

Characteristics are described using descriptive statistics. Frequencies and percentages are used to describe categorical variables. Means and SDs (or medians and ranges where appropriate) are used to describe continuous variables. Chi-square test was used to test for associations in bivariate comparisons. Wilcoxon rank-sum test was used to test for differences in continuous variables among the 3 troponin categories. Logistic regression using the Firth penalized likelihood approach was used to test the predictive ability of variables of interest. Multinomial generalized logistic regression was used to test the predictive ability of variables of interest for categorical outcomes with more than 2 levels. Nonparametric comparisons using the method described by DeLong et al were performed to assess differences in receiver operating characteristic (ROC) curves.^9^ A value of 0.0001 was used to assess statistical significance. All statistical analyses were performed in SAS statistical software, version 9.4 (SAS Institute Inc).

## RESULTS

Baseline characteristics of this patient population are presented in Table 1. The median age of patients who presented to the hospital was 70 years (interquartile range [IQR] 60-81 years), with an age range of 40 to 104 years old. Hypertension was the most common risk factor in the study population (78.81% of patients), and coronary artery disease was the most common history (30.09% of patients).

**Table 1.** Baseline Characteristics of Patients With at Least One Troponin Value and Age ≥40 Years

Variable	Patient Population, n=30,173
Demographics	
Age, years, median (range)	70 (40-104)
Sex	
Male	14,497 (48.05)
Female	15,676 (51.95)
Risk factors	
Smoker	6,069 (20.11)
Hypertension	23,780 (78.81)
Hyperlipidemia	16,464 (54.57)
Type 1 diabetes mellitus	199 (0.66)
Type 2 diabetes mellitus	11,229 (37.22)
History	
Coronary artery disease	9,079 (30.09)
Chronic kidney disease	6,011 (19.92)
Chronic obstructive pulmonary disease	5,899 (19.55)
Prior cerebral vascular accident	724 (2.40)
Prior myocardial infarction	2,531 (8.39)

Note: Data are presented as n (%) unless otherwise noted.

Among the study population, the median initial troponin was 0.02 ng/dL (IQR 0.01-0.07 ng/dL), with a range of 0 to 360.92 ng/dL (reference range, 0.00-0.09 ng/dL). The median maximum troponin was 0.03 ng/dL (IQR 0.01-0.10 ng/dL), with a range of 0 to 647.54 ng/dL, and the median change in troponin was 0.00 ng/dL (IQR 0.00-0.03 ng/gL) with a range of 0 to 647.54 ng/dL (Table 2).

**Table 2.** Quartile Distribution of Initial, Maximum, and Change in Troponin Level

Troponin Level	n	25th Percentile	Median (range)	75th Percentile
Initial troponin	22,384^a^	0.01	0.02 (0-360.92)	0.07
Maximum troponin	23,965	0.01	0.03 (0-647.54)	0.10
Change in troponin (maximum – minimum)	23,965	0.00	0.00 (0-647.54)	0.03

^a^In our analysis, an initial troponin value that was below detectable limit was considered missing; hence, the n is 1,581 less than the sample size for the maximum troponin and change in troponin.

**Figure 1.**
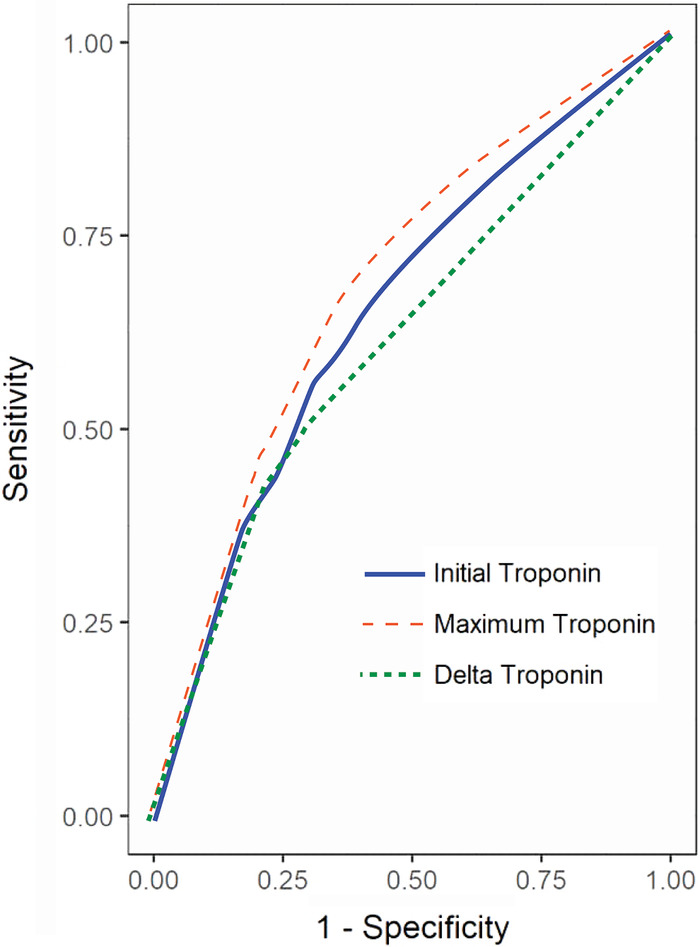
Receiver operating characteristic (ROC) curve comparison for mortality. The univariate ROC curves for initial, change, and maximum troponin are overlaid. The curves graph the true positive (sensitivity) rate by the false negative rate (1-specificity); curves closer to the top right corner indicate better diagnostic performance. Compared to initial and delta troponin values (area under the curve [AUC] 0.65 and 0.61, respectively), maximum troponin (AUC 0.68) best predicts mortality.

Troponin as a significant predictor of death was analyzed showing that the odds of death increase by 0.9% (odds ratio [OR] 1.009, 95% CI 1.004-1.013) for each unit of increase of the initial troponin. Likewise, the odds of death increase by 0.7% (OR 1.007, 95% CI 1.005-1.009) for each unit of increase in both the maximum and change in troponin (Table 3). A ROC curve demonstrated that the area under the curve (AUC) for maximum troponin level is 0.6782 (95% CI 0.6576-0.6989), which represented a statistically significant ability to predict death when compared to the AUC for the initial 0.6547 (95% CI 0.6334-0.6760) and the AUC for the change in troponin 0.6113 (95% CI 0.5892-0.6333) levels (Table 3, Figure 1). When comparing the ROC curve for maximum vs initial, change vs initial, and change vs maximum, the C-statistic for maximum troponin is larger than the initial or change in troponin, which represents a statistically significant improvement in predictive ability for maximum troponin to predict mortality (Table 4).

**Table 3.** Logistic Regression With the Firth Penalized Likelihood Approach for Initial, Maximum, and Change in Troponin on Mortality

Troponin Level	Odds Ratio	95% CI	*P* Value	AUC	95% CI
Initial troponin	1.009	1.004-1.013	0.0002	0.6547	0.6334-0.6760
Maximum troponin	1.007	1.005-1.009	<0.0001	0.6782	0.6576-0.6989
Change in troponin (maximum – minimum)	1.007	1.005-1.009	<0.0001	0.6113	0.5892-0.6333

Note: Logistic regression with the Firth penalized likelihood approach was performed to assess the predictive ability of initial, maximum, and change in troponin level on death at index admission. The *P* value for troponin value indicates statistical significance for predicting mortality.

AUC, area under the receiver operating characteristic curve.

**Table 4.** Receiver Operating Characteristic (ROC) Curve Comparisons for Initial, Maximum, and Change in Troponin on Mortality

Troponin Comparison	Difference in ROC Curves	95% Wald Confidence Limits	*P* Value
Maximum vs initial	0.0235	0.0119 to 0.0352	<0.0001
Change vs initial	–0.0434	–0.0670 to –0.0198	0.0003
Change vs maximum	–0.0670	–0.0839 to –0.0500	<0.0001

Note: Nonparametric comparisons of area under the ROC curves were performed to test for significant differences in the ability of each univariate model to predict mortality. The maximum troponin value model predicts death significantly better than the initial troponin model and the change in troponin model.

**Figure 2.**
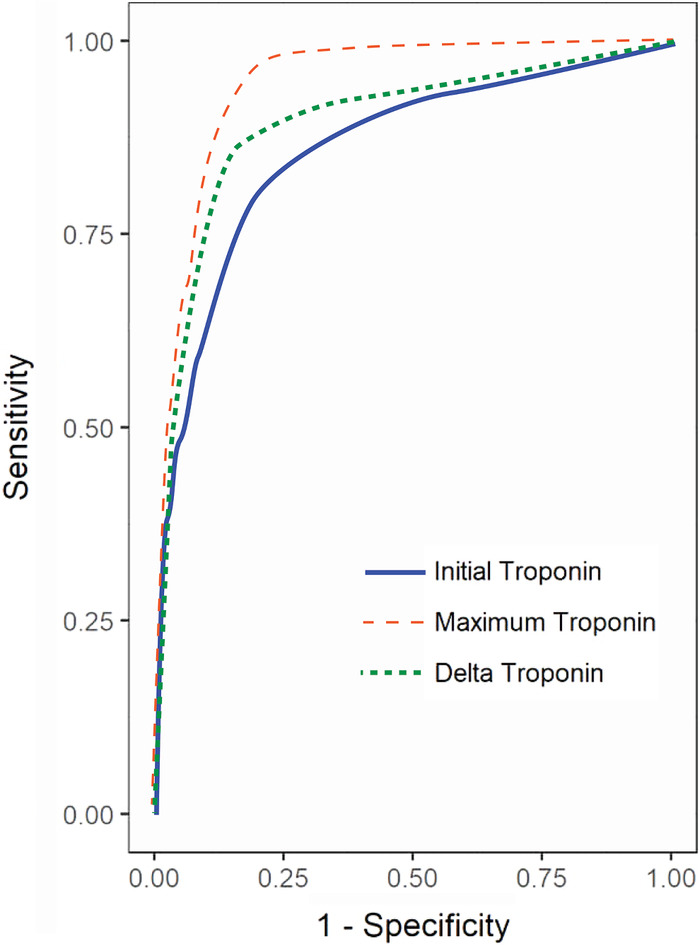
Receiver operating characteristic (ROC) curve comparison for acute myocardial infarction diagnosis. The univariate ROC curves for initial, change, and maximum troponin are overlaid. The curves graph the true positive (sensitivity) rate by the false negative rate (1-specificity); curves closer to the top right corner indicate better diagnostic performance. Compared to initial and delta troponin values (area under the curve [AUC] 0.86 and 0.89, respectively), maximum troponin (AUC 0.94) best predicts acute myocardial infarction.

A secondary analysis of initial troponin as a significant predictor of diagnosis of AMI vs other causes of troponin elevation showed that the odds of AMI increased by 7.4% for each unit of increase of the initial troponin value (OR 1.074, 95% CI 1.063-1.086) (Table 5). Similarly, troponin as a significant predictor of AMI for maximum and change in troponin vs other causes showed the odds of AMI increased by 3.1% for each unit of increase of the maximum and change in troponin value (OR 1.031, 95% CI 1.028-1.034 and OR 1.031, 95% CI 1.027-1.034, respectively) (Table 5). Similarly, the AUC for maximum troponin level 0.9401 (95% CI 0.9353-0.9449) showed a statistically significant ability to predict an AMI compared to the AUC for initial troponin 0.8562 (95% CI 0.8454-0.8670) and the AUC for the change in troponin 0.8939 (95% CI 0.8848-0.9030) levels (Table 5, Figure 2). When comparing the ROC curve for maximum vs initial, change vs initial, and change vs maximum, the C-statistic for maximum troponin is larger than the initial or change in troponin, which represents a statistically significant improvement in predictive ability for maximum troponin to predict AMI (Table 6).

**Table 5.** Logistic Regression With the Firth Penalized Likelihood Approach for Initial, Maximum, and Change in Troponin on Acute Myocardial Infarction

Troponin Level	Odds Ratio	95% CI	*P* Value	AUC	95% CI
Initial troponin	1.074	1.063-1.086	<0.0001	0.8562	0.8454-0.8670
Maximum troponin	1.031	1.028-1.034	<0.0001	0.9401	0.9353-0.9449
Change in troponin (maximum – minimum)	1.031	1.027-1.034	<0.0001	0.8939	0.8848-0.9030

Note: Logistic regression with the Firth penalized likelihood approach was performed to assess the predictive ability of initial, maximum, and the change in troponin level on a primary diagnosis of acute myocardial infarction vs any other diagnosis. The *P* value for troponin value indicates statistical significance for predicting acute myocardial infarction.

AUC, area under the receiver operating characteristic curve.

**Table 6.** Receiver Operating Characteristic (ROC) Curve Comparisons for Initial, Maximum, and Change in Troponin on Acute Myocardial Infarction

Troponin Comparison	Difference in ROC Curves	95% Wald Confidence Limits	*P* Value
Maximum vs initial	0.0839	0.0737 to 0.0941	<0.0001
Change vs initial	0.0377	0.0237 to 0.0517	<0.0001
Change vs maximum	–0.0462	–0.0540 to –0.0385	<0.0001

Note: Nonparametric comparisons of area under the ROC curves were performed to test for significant differences in the ability of each univariate model to predict acute myocardial infarction. The maximum troponin value model predicts acute myocardial infarction significantly better than the initial troponin model and the change in troponin model.

When comparing the initial, maximum, and change in troponin levels for prediction of AMI, all 3 levels are statistically significant predictors of AMI vs other cardiac causes (non-AMI), pulmonary, sepsis, or others (all other categories) (Table 7). When initial, maximum, and change in troponin levels are used for prediction of AMI vs other cardiac causes (non-AMI), pulmonary, sepsis, or others (all other categories), maximum troponin level had the highest discriminatory ability for an AMI diagnosis compared to the initial or change in troponin level (C-statistic=0.6391) (Table 8).

**Table 7.** Multinominal Logistic Regression for Initial, Maximum, and Change in Troponin to Predict Acute Myocardial Infarction (AMI) vs Non-AMI Cardiac, Pulmonary, Sepsis, and Other Diagnoses

Comparison	Troponin Level	Odds Ratio	95% CI	*P* Value
AMI vs non-AMI cardiac	Initial	1.042	1.033-1.053	<0.0001
	Maximum	1.018	1.015-1.020	<0.0001
	Change	1.018	1.015-1.020	<0.0001
AMI vs pulmonary	Initial	2.364	1.852-3.165	<0.0001
	Maximum	1.468	1.330-1.653	<0.0001
	Change	1.471	1.314-1.692	<0.0001
AMI vs sepsis	Initial	1.063	1.047-1.082	<0.0001
	Maximum	1.035	1.029-1.042	<0.0001
	Change	1.035	1.028-1.042	<0.0001
AMI vs other diagnoses	Initial	1.335	1.285-1.391	<0.0001
	Maximum	1.122	1.109-1.136	<0.0001
	Change	1.118	1.103-1.133	<0.0001

**Table 8.** Multinominal Area Under the Curve for Initial, Maximum, and Change in Troponin

Troponin Level	C-Statistic
Initial	0.6061
Maximum	0.6391
Change	0.6271

Note: The multinominal areas under the curve show that the maximum troponin level is most predictive of acute myocardial infarction vs other diagnoses.

## DISCUSSION

The ability to detect minute cardiomyocyte damage has resulted in a large burden to the health care system as a positive troponin value often results in an obligatory stay in the hospital to rule out an AMI, which is not a cost-effective solution but may be a necessary part of the workup process. In our study, we demonstrated that the maximum troponin level, compared to the initial level or the change in troponin level, resulted in the best detection of AMI and was most predictive of mortality. A change in troponin level during a 3- or 6-hour window, depending on conventional or sensitive assay, may not safely rule out AMI. Cardiac-specific troponins I and T are “organ-specific, but not disease-specific.”^1,3^ The evolution of cardiac-specific troponins I and T from conventional to high-sensitivity troponins has presented a clinical conundrum as these troponins are often elevated above the 99th percentile in non-AMI conditions.^1-4,10^ The diagnostic utility of high-sensitivity cardiac troponins for AMI is dependent on the troponin level, which corresponds with the amount of cardiomyocyte injury.^1,2,5^ The ability to risk stratify is paramount in identifying patients with AMI vs other conditions. In several large prospective diagnostic studies, the absolute change consistently provided significantly higher diagnostic accuracy than the relative changes to distinguish AMI from non-AMI causes.^1^

Regardless of the cause, an elevated cardiac troponin level is associated with a 4.5% 30-day risk of major adverse events defined as all-cause mortality and nonfatal outcomes.^1,2,5^ The development of highly sensitive cardiac troponin assays allows for faster risk stratification of individuals at high risk for AMI. However, a positive troponin value also leaves a large proportion of patients who will leave the hospital without a specific diagnosis, which can ultimately lead to significant morbidity and mortality.^1,4,7^ Our study indicates that the maximum troponin level is a more sensitive and specific predictor of mortality than the initial troponin level or change in troponin level. We hypothesize that maximum troponin is the best predictor of AMI because of the correlation of elevations in cardiac troponins to the amount of cardiac damage. Given the significant burden of troponin testing in the inpatient setting, using our model for every unit of increase in troponin signifies an increase in the odds of death by 0.7%. Therefore, individuals with significant elevations in cardiac troponins should have further diagnostic studies to identify a cause before discharge from the hospital. The optimal treatment for these patients is not known, and the mitigation of future risk of morbidity and mortality needs further study.^11^

Given the high sensitivity and specificity of cardiac troponins (>50% of the general population with chest pain will have a cardiac troponin above the 99th percentile), they have clinical uses for both ruling in and ruling out AMI. However, cardiac troponins need to be ordered and interpreted in the right clinical context to achieve the intended purpose.^3,10,12^ Elevations in cardiac troponins are seen in many non-AMI conditions such as sepsis, pulmonary embolism, heart failure, chronic kidney disease, stroke, hypertension or hypotension, and liver failure, making differentiating AMI from non-AMI conditions difficult for clinicians.^2,4,5^ Both AMI and non-AMI conditions can cause elevations in cardiac troponins that result in an increase in mortality regardless of the troponin level.^2,3,4,5,10^ Our study indicates that maximum troponin level is better able to distinguish between AMI and non-AMI conditions than initial troponin or change in troponin level, with 3.1% greater odds of diagnosing AMI for each unit of increase of maximum troponin. Clinically, this finding can help distinguish between different diagnostic studies and interventions needed for AMI and non-AMI conditions.

Our study has several limitations. First, a retrospective analysis using electronic medical records introduces inherent biases; however, this bias was mitigated as we considered all cardiac troponins drawn in a 2-year period. Second, this study was conducted in a 9-hospital system in Texas, which may not be applicable to other populations across America.

## CONCLUSION

Our study demonstrates that maximum troponin level rather than initial or change in troponin is most predictive of mortality and AMI. The majority of elevated troponin levels do not represent AMI but rather a non-AMI condition. However, regardless of the troponin level, an increase in mortality was observed in the population. More studies need to be performed to determine the clinical significance of maximum troponin levels in clinical practice.

## Notes

*This article meets the Accreditation Council for Graduate Medical Education and the American Board of Medical Specialties Maintenance of Certification competencies for Patient Care, Medical Knowledge, Systems-Based Practice, and Practice-Based Learning and Improvement.*
